# Mucoadhesive Buccal Films for Local Delivery of *Lactobacillus brevis*

**DOI:** 10.3390/pharmaceutics12030241

**Published:** 2020-03-08

**Authors:** Angela Abruzzo, Beatrice Vitali, Francesca Lombardi, Luca Guerrini, Benedetta Cinque, Carola Parolin, Federica Bigucci, Teresa Cerchiara, Catia Arbizzani, Maria Caterina Gallucci, Barbara Luppi

**Affiliations:** 1 Department of Pharmacy and Biotechnology, University of Bologna, Via San Donato 19/2, 40127 Bologna, Italyb.vitali@unibo.it (B.V.); carola.parolin@unibo.it (C.P.); federica.bigucci@unibo.it (F.B.); teresa.cerchiara2@unibo.it (T.C.); 2 Department of Life, Health and Environmental Sciences, University of L’Aquila, Via Pompeo Spennati, Building Rita Levi Montalcini, Coppito, 67100 L’Aquila, Italy; francesca.lombardi@univaq.it (F.L.); luca.guerrini@graduate.univaq.it (L.G.); benedetta.cinque@univaq.it (B.C.); 3 Department of Chemistry “Ciamician”, University of Bologna, Via Selmi 2, 40126 Bologna, Italy; catia.arbizzani@unibo.it; 4 Department of Chemistry and Chemical Technology, Calabria University, Arcavacata di Rende, Via P. Bucci, Cubo 15D, 87036 Cosenza, Italy; cate75@gmail.com

**Keywords:** *Lactobacillus brevis* CD2, arginine deiminase activity, buccal films, hydroxypropylmethylcellulose, mucoadhesion

## Abstract

The aim of this work was to prepare mucoadhesive buccal films for local release of *Lactobacillus brevis* CD2, which shows interesting anti-inflammatory properties due to its high levels of arginine deiminase. Hydroxypropylmethylcellulose-based films were prepared by means of a modified casting method, which allowed *L. brevis* CD2 loading on one side of the film, before its complete drying. Three batches of films were prepared, stored at +2–8 °C and +23–25 °C for 48 weeks and characterized in terms of physico-chemical and functional properties. For each batch, the *L. brevis* viable count and arginine deiminase activity were evaluated at different time points in order to assess functional property maintenance over time. Moreover, the mucoadhesive properties and ability of the films to release *L. brevis* CD2 were evaluated. A good survival of *L. brevis* CD2 was observed, particularly at the storage temperature of +2–8 °C, while the activity of arginine deiminase was maintained at both temperature values. Films showed good mucoadhesive properties and guaranteed a prolonged release of viable lactobacilli, which can be directed towards the whole buccal cavity or specific mucosa lesions. In conclusion, the proposed preparative method can be successfully employed for the production of buccal films able to release viable *L. brevis* CD2 cells that maintain the anti-inflammatory enzymatic activity.

## 1. Introduction

The oral cavity hosts a complex microbiota, of which dental plaque is the major physiological component. Oral microbiota represent a host defense system, able to prevent the colonization of other pathogenic microorganisms and remain relatively stable over time, despite regular environmental changes [1,2,3]. When an imbalance appears among the indigenous bacteria, oral pathologies, such as dental caries, periodontal disease and halitosis, could occur [4]. Dental caries is characterized by overproliferation of *Streptococcus mutans* [2,5]; while periodontal diseases, such as periodontitis, are associated with *Porphyromonas gingivalis, Treponema denticola, Tannerella forsythia* and *Aggregatibacter actinomycetemcomitans*, which can colonize the subgingival sites, escape the host’s defense system and cause tissue damage [6,7]. Another important problem connected with the buccal cavity is oral mucositis, which can appear following chemotherapy and radiotherapy. Mucositis is widely associated with bacterial and viral infections and leads to consequences that may limit the adequate nutritional intake and impair the patient’s quality of life [8,9]. Although the pathogenesis of these diseases is not completely understood, several factors are believed to play an important role, including inflammatory cytokines [8,10], prostaglandin E2 (PGE2) [11], nitric oxide (NO) [12,13] and salivary immunoglobulin-A (IgA) [14].

The use of probiotics could represent an alternative and a promising way to limit the growth of pathogenic members in the buccal environment, thus improving dental and buccal health. In fact, probiotic bacteria secrete various antimicrobial substances such as organic acids, hydrogen peroxide and bacteriocins and compete with pathogenic microorganisms for adhesion sites on the mucosa [15,16]. Probiotics can also modify the surrounding environment by modulating the pH and/or the oxidation–reduction potential, which may compromise pathogens’ stability [15]. Finally, probiotics may provide beneficial effects by stimulating nonspecific immunity and modulating the humoral and cellular immune response [17,18]. 

Some authors have studied the beneficial effects of *Lactobacillus brevis* CD2 in the treatment of oral pathologies. Della Riccia and colleagues [19] evaluated the anti-inflammatory effects of this strain in a group of patients with chronic periodontitis and observed a significant reduction in salivary levels of PGE2, nitrite/nitrate, pro-inflammatory cytokine IFN-γ, and matrix metalloproteinases (MMPs). *L. brevis* was also proposed as a promising probiotic strain able to prevent the biofilm formation of *Prevotella melaninogenica,* a well-known causative agent of periodontitis [20]. The strain *L. brevis* CD2 was shown to have high levels of arginine deiminase and sphingomyelinase [21]. Eukaryotic human cells can convert arginine into nitric oxide and polyamines by the action of nitric oxide synthase and arginase, respectively. Arginine deiminase of bacterial origin competes with nitric oxide synthase and converts arginine to ammonia and citrulline, down-regulating arginine conversion to nitric oxide [21] and, consequently, leading to a reduction in the levels of some of the known inflammatory parameters (cytokines IL-1a, IL-6, IL-8, TNF-a, IFN-γ, PGE2 and MMPs). Moreover, bacterial sphingomyelinase can hydrolyze the platelet activating factor (PAF), a potent inflammatory cytokine, known to be associated with oral mucositis in radiation therapy [22,23]. The efficacy of lozenges containing *L. brevis* CD2 was also tested in the treatment of oral ulcers in Behcet’s syndrome patients, and a significant decrease in the number of oral ulcers after 1 and 2 weeks of therapy was observed [24]. Finally, the study of Campus and colleagues demonstrated the ability of *L. brevis* CD2 lozenges to reduce plaque acidogenicity, salivary *S. mutans* and bleeding on probing [25].

For the treatment of oral cavity pathologies, the selection of an appropriate delivery system is a key factor for the therapy success. In particular, solid systems based on mucoadhesive polymers can be retained inside the buccal cavity, thus increasing the contact time of the formulation with the buccal mucosa [26,27]. Among the different solid dosage forms, films are considered suitable formulations in terms of comfort thanks to their thin thickness and easy application [28]. Regarding the delivery of probiotic species in the buccal cavity for the treatment of oral pathologies, bacterial survival during the technological process and the product shelf life is an important pre-requisite in the development of a formulation strategy. The present study aims to develop a solid dosage form, alternative to tablets and chewing gums, capable of modulating the oral microbiota. Oral thin films for the fast release of *Lactobacillus fermentum* NCIMB 5221, a probiotic bacterium with remarkable antioxidant capability, have been investigated by Saha and colleagues for the treatment and prevention of oral disorders [29]. The authors prepared carboxymethyl cellulose films starting from the suspension of an overnight bacterial culture in the polymeric solution and using a classical casting method performed by drying at room temperature. Their results showed successful maintenance of the anti-inflammatory activity of the probiotic formulation for 150 days, and a significant loss in bacterial viability was observed after this time point. For this reason, they suggested the use of an optimized drying apparatus and the addition of a protectant, such as a sugar or a prebiotic, to prolong the probiotic viability. Heinemann and coauthors [30] formulated orally-disintegrating films for the delivery of *Lactobacillus acidophilus* or *Bifidobacterium animalis* subsp. *lactis*, which were entrapped in a matrix composed of carboxymethyl cellulose, gelatin and starch. In their study, bacterial counts showed less than 15% loss of probiotics during the production process, but probiotics were viable only for 90 days of storage. 

The aim of this study was to obtain a mucoadhesive buccal film able to deliver *L. brevis* CD2 and their metabolites inside the buccal cavity or towards the mucosa by just changing the application side, according to the desired local treatment (e.g., oral mucositis and ulcers or oral cavity pathologies, such as the chronic periodontitis). The technological challenge was the development of a productive strategy able to guarantee the maintenance of the functional properties of the probiotic-based film for a long storage period. In this perspective, hydroxypropylmethylcellulose films were prepared by means of a modified casting method, which permitted us to load freeze-dried *L. brevis* CD2 on one face of the film. Films were characterized in terms of physico-chemical and functional properties. The viable counts of the probiotic strain and arginine deiminase activity were evaluated at different time points until 48 weeks, in order to investigate the probiotic survival and biological activity over time. Finally, in vitro mucoadhesion and release studies were performed to evaluate film ability to adhere to buccal mucosa and to release *L. brevis* CD2, respectively.

## 2. Materials and Methods

### 2.1. Materials

Hydroxypropylmethylcellulose (HPMC, Methocel K100) and propylene glycol (PG) were obtained from Fluka (Milan, Italy). Samples of *L. brevis* CD2 were obtained from Prof. M.G. Cifone (Department of Life, Health and Environmental Sciences, University of L’Aquila, L’Aquila, Italy) in a pure lyophilized form [(10^11^ colony-forming units per gram (CFU/g)]. MRS, Nutrient Agar (NA) and Sabouraud Dextrose Agar (SDA) were obtained commercially from Difco (Becton, Dickinson and Co., Sparks, MD, USA); Anaerocult A was purchased from Merck KGaA (Darmstadt, Germany). l-Citrulline, l-Arginine and 2,3-butanedione monoxime were purchased from Sigma (St. Louis, MO, USA). All other chemicals and solvents were of analytical grade and purchased from Carlo Erba (Milan, Italy). Water-uptake, mucoadhesion and release studies were carried out in aqueous buffer at pH 6.8, simulating human saliva pH, composed of 4.61 g/L KH_2_PO_4_ and 16.75 g/L Na_2_HPO_4_·12H_2_O (healthy saliva pH = 6.7–7.4 [31]). 

### 2.2. Preparation of HPMC Buccal Films

Films were prepared through the modified casting-solvent evaporation method. HPMC (2.5% *w/w*) was solubilized in water containing PG (1% *w/w*) and the solution was stirred for 8 h until a viscous, gel-like solution was formed. The viscosity (rotational viscometer, +23–25 °C, spindle TR11, RPM 50, Visco Star, Fungilab S.A., Barcelona, Spain) and the pH (pH meter, MicroPH CRISON 2000, Modena, Italy) of the polymeric gel-like solution were 9100 mPa·s and 6.3, respectively. Then the viscous solution was left overnight at room temperature to ensure a clear, bubble-free gel. The bubble-free gel (6.7 g) was casted on a Petri dish (5 cm in diameter) and oven-dried at 50 °C for 5 h (heating oven FD series; Binder, Tuttlingen, Germany). After this time, *L. brevis* CD2 was loaded by means of a capsule-based dry powder dispenser onto the exposed face of the film (loaded face). Glass rings of suitable size (height, 1 cm; diameter, 1 cm) were used in order to confine an adequate amount (10 mg, corresponding to 9.21 ± 0.25 log CFU) of bacterial cells and cut the film into the final dosage forms. Finally, the Petri dish was placed in a desiccator at 20 °C for 24 h in order to complete the film formation (until constant weight). 

Three batches of films were produced. Films were stored in a well-closed container at two different temperatures (+2–8 °C and +23–25 °C) for 48 weeks. Probiotic viability and arginine deiminase activity were evaluated at different time points: time zero (t0), 2 weeks (t1), 4 weeks (t2), 8 weeks (t3), 12 weeks (t4), 24 weeks (t5), 36 weeks (t6) and 48 weeks (t7). 

### 2.3. Characterization of Buccal Films 

The average thickness (Mitutoyo pocket thickness gauge; Mitutoyo Mfc. Co. Ltd, Tokyo, Japan) and the average weight (electronic balance) were determined from the observation of three films in each batch. Thickness uniformity was evaluated taking observations at five different random points on one film of each batch. Thermogravimetric analysis was performed using TA Instruments Q50 (β = 10 K min^−1^, argon gas flow rate 60 mL/min, 30–100 °C temperature range and isothermal at 100 °C for 15 min) in order to evaluate the water content. Measurements were carried out on three films for each batch.

### 2.4. Scanning Electron Microscopy (SEM)

SEM analyses were performed in order to study the morphological structure of buccal films. Films were fixed on supports and coated with gold-palladium under an argon atmosphere using a gold sputter module in a high-vacuum evaporator. Samples were then observed by LEO 420 (LEO Electron Microscopy Ltd., Cambridge, UK) using secondary electron imaging at 1.5 kV in order to examine film surfaces.

### 2.5. In Vitro Water-Uptake Studies 

In vitro water-uptake studies were performed in phosphate buffer at pH 6.8, simulating human saliva pH [31], by measuring the increase of weight at predetermined time points [26]. Films were weighed and placed with filter papers (diameter 40 mm) on the top of a sponge (5 cm × 5 cm × 2 cm) previously soaked in the hydration medium inside a Petri dish filled with the same buffer to a height of 0.5 cm. Water uptake was determined as weight increase of the film after 6 h, according to the following equation:

(1) $$\%Water\;Uptake\;\left(WU\right)=\;\frac{\left(W_{\mathrm{Hfp}}-W_{\mathrm{Hp}}\right)}{W_{\mathrm{Df}}}$$

where *W*_Hfp_ is the weight of the hydrated film and wet filter paper, *W*_Hp_ is the weight of wet filter paper and *W*_Df_ is the initial weight of the dry film. Measurements were carried out on three films for each batch.

### 2.6. Microbiological Analysis

The viable count of *L. brevis* CD2 in HPMC films, stored at +2–8 °C and +23–25 °C, was evaluated at the different time points of the study (t0, t1, t2, t3, t4, t5, t6 and t7). The buccal film was suspended in 10 mL of phosphate buffer (pH 6.8) containing 0.05% of l-cysteine. Incubation at 37 °C for 24 h provided the complete dissolution of the film. Serial dilutions, prepared in saline, were spread onto MRS agar plates containing 0.05% of l-cysteine for the selective count of *L. brevis* CD2. Plates were incubated at 37 °C for 48 h under anaerobic conditions, using anaerobic jars and Anaerocult A (Merck, Milan, Italy). Moreover, in order to evaluate microbial contamination of films, the same serial dilutions were spread onto NA and SDA plates for the count of total bacteria and fungi, respectively. Plates were incubated in aerobic conditions at 37 °C (NA) and 30 °C (SDA) for 48 h. Plate counts were performed in duplicate. The results obtained were converted to logarithmic values and the means and standard deviations were calculated.

### 2.7. Arginine Deiminase Activity In Buccal Films

The buccal film was resuspended in 1 mL di NaCl 0.9M and sonicated in ice (30 min, alternating 10 s sonication and 10 s pause) using a Vibracell sonicator (Sonic and Materials, Danbury, CT, USA) (bacterial film crude extract). The assay for arginine deiminase activity was based on the method of Zúñiga and coauthors [32] with some modifications. Briefly, arginine deiminase was assayed in a reaction mixture containing 10 mM phosphate buffer (pH 7.0), 3 mM L-arginine and the specific volume of bacterial film crude extract (50 µL). After incubation at 37 °C for 1 h the reaction was stopped by the addition of 2 M HCl, and samples were centrifuged at 11,000× *g* for 20 min. The formed citrulline was measured as previously reported [33]. To 1 mL of the centrifuged sample, 1.5 mL of an acid mixture (H_2_SO_4_–H_3_PO_4_; 1:3, *v/v*) and 0.25 mL of 1.5% diacetyl monoxime (2,3-butanedione monoxime) were added, mixed together, and boiled in the dark for 30 min. After cooling in the dark for 10 min, the absorbance of samples and citrulline standards (made in the reaction mixture and treated in the same way) was measured at 490 nm. The hydrolysis of arginine in the buccal film was expressed as µmol citrulline/h/film and determined by comparison to the standard curve.

### 2.8. In Vitro Residence Time and Mucoadhesive Ability of Buccal Films

Film mucoadhesive properties were investigated in terms of residence time on a freshly excised mucosa and ability to adhere to the mucosa. For this study, freshly isolated porcine esophageal mucosa was employed on the basis of its similarity to the buccal one [34]. The mucosa was isolated from pig esophageal tissue, purchased from a local slaughterhouse, following the procedure described in our previous work [35]. For the evaluation of in vitro residence time, the mucosa (surface area of 4 cm^2^) was pasted on a glass slide using cyanoacrylate adhesive and hydrated with 20 µL of phosphate buffer at pH 6.8 for 5 min. Then, the film was attached to the mucosa with a slight pressure for 2 min and the ensemble was immersed inside a beaker containing 20 mL of phosphate buffer at pH 6.8. Films were attached with the loaded face towards the mucosa or medium. The time taken by the film for complete detachment from the mucosa was considered as the residence time [36]. Mucoadhesive ability was measured in terms of the force needed to pull out the mucosa from the film with an adapted tensiometer (Krüss 132869; Hamburg, Germany) [26,35]. The mucosa was hydrated for 5 min with phosphate buffer at pH 6.8, fixed to a support (surface area 1 mm^2^) with cyanoacrylate adhesive and then suspended from the tensiometer. Subsequently, the mucosa was lowered to make contact with the loaded or unloaded surface of the film placed on a glass slide. A 20 × 10^−5^ N force, measured by the torsion balance of the instrument as a negative force, was applied to the film for 60 s. The adhesive bond strength was represented by the force (reported in Newtons), required to separate the mucosa from the film.

### 2.9. In Vitro Release of L. Brevis

For this study, the film was attached to a glass slide using cyanoacrylate adhesive and then placed in 20 mL of phosphate buffer at pH 6.8 under stirring (50 rpm) for 360 min. Films were attached facing the loaded face towards the glass slide or the buffer, in order to evaluate the amount of lattobacilli released over time from the different film faces [35]. Samples were taken at predetermined time points, replaced by fresh medium and analyzed by spectroscopy at 600 nm. Moreover, the viable count of *L. brevis* CD2 released from HPMC films after 360 min was evaluated following the procedure reported in Section 2.6.

### 2.10. Statistical Analysis

Results are expressed as mean ± SD. The t-test and two-way ANOVA were used to determine statistical significance, by using GraphPad software (GraphPad Prism 8.0 version for Windows, GraphPad Software, San Diego, California, USA, www.graphpad.com). Differences were considered to be significant for values of *p* < 0.05.

## 3. Results

### 3.1. Characterization of Buccal Films

Films of 1 cm diameter with a weight in the range of 9.0 mg to 9.6 mg were obtained through the modified casting-solvent evaporation method. The film thickness varied from 80 ± 6 µm to 88 ± 5 µm; the thickness was found to be uniform (*p* > 0.05, Table 1). The storage conditions did not affect the film weight and thickness, which were maintained over time (data not reported).

Water content % were 5.3 ± 0.3, 5.0 ± 0.2 and 5.4 ± 0.2% for batch I, II and III, respectively. No significant difference was observed between the three batches (*p* > 0.05). 

### 3.2. Scanning Electron Microscopy (SEM)

**Figure 1:**
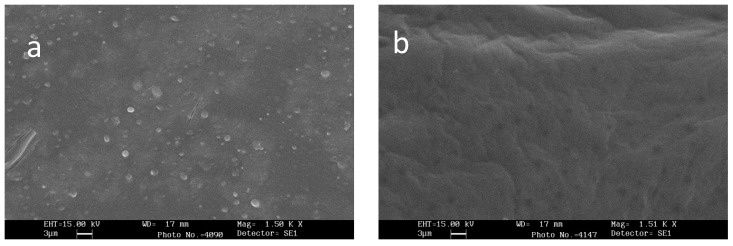
Scanning electron micrographs of films loaded with *L. brevis* CD2: (**a**) top surface and (**b**) bottom surface.

SEM images showed homogenous distribution of lactobacilli on the film top surface, on which bacteria were nebulized (Figure 1a), while the film bottom surface was smooth and did not show the presence of bacteria (Figure 1b).

### 3.3. In Vitro Water-Uptake Studies

**Figure 2:**
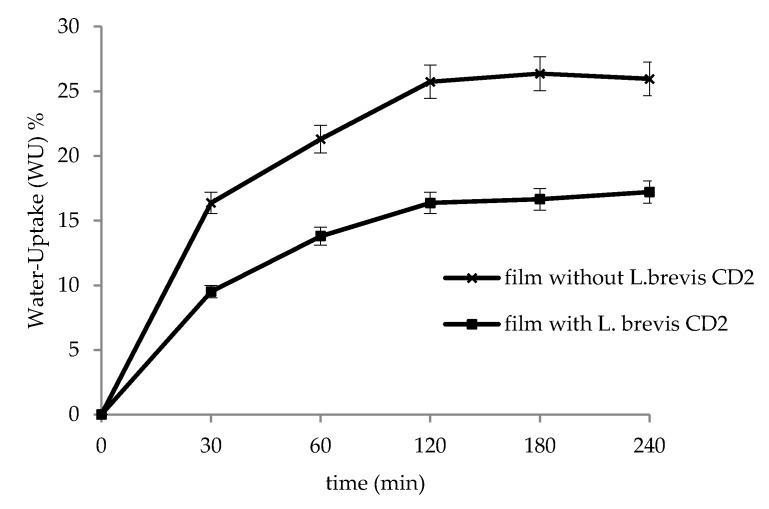
Water-uptake ability of unloaded films and *L. brevis* loaded films. For each batch, three determinations were carried out. Data reported correspond to the mean of five measurements ± standard deviation.

Figure 2 shows the in vitro water-uptake results, obtained at pH 6.8 on unloaded HPMC films and probiotic-loaded films. Films showed good ability to hydrate and the major water-uptake % was found at the time point of 120 min, when the films started to create a semisolid system (gel-like structure). Moreover, unloaded films showed greater water uptake capacity with respect to films containing the probiotic strain (*p* < 0.05). This behavior can be attributed to the peptidoglycan layer of *L. brevis* CD2, made up of a series of sugars, which can physically interact with HPMC, thus reducing its ability to hydrate. The ability of sugars to limit HPMC hydration was in agreement with other observations reported in the literature [37,38].

### 3.4. Bacterial Survival and Film Microbial Contamination

**Figure 3:**
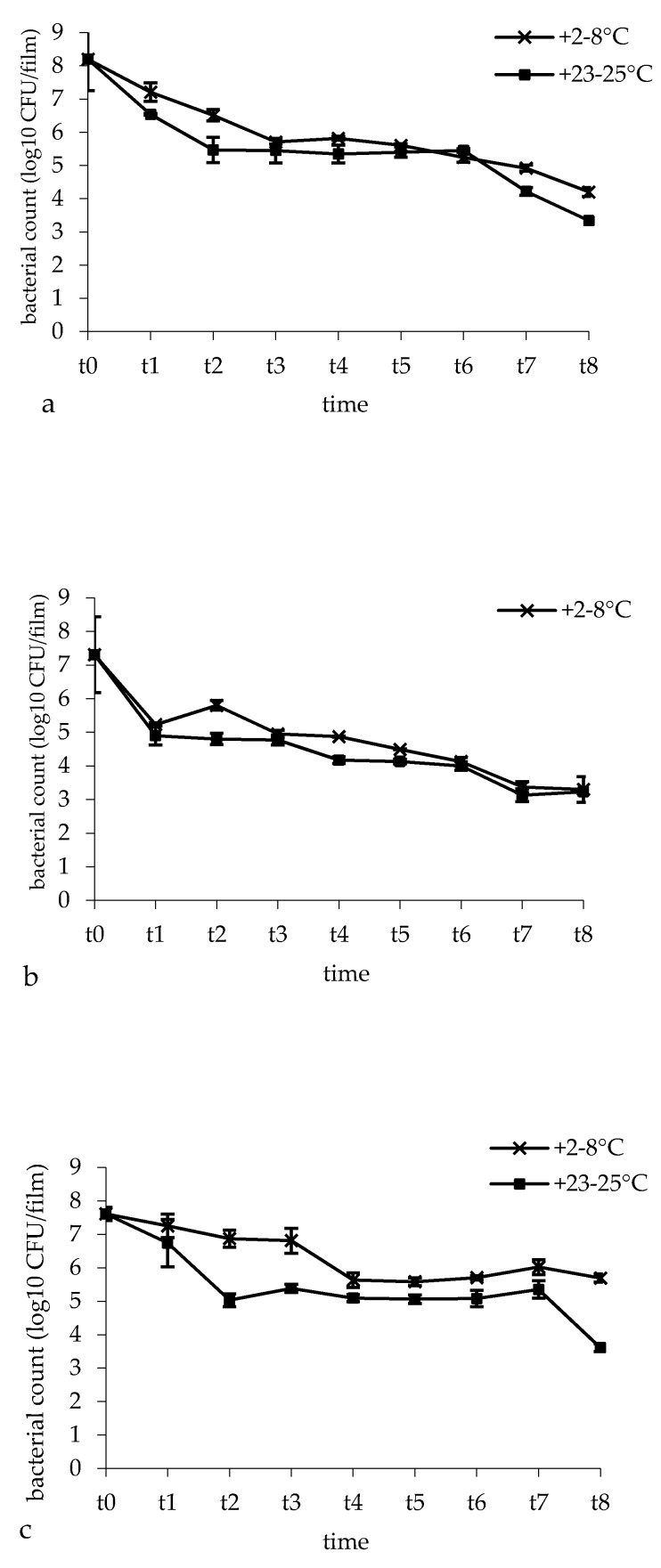
*L. brevis* CD2 survival in the buccal films during a storage period of 48 weeks at the temperatures of +2–8 °C and +23–25 °C. t0: time zero; t1: 2 weeks; t2: 4 weeks; t3: 8 weeks, t4: 12 weeks; t5: 24 weeks; t6: 36 weeks; t7: 48 weeks. (**a**) Batch I; (**b**) batch II; (**c**) batch III. Data are expressed as mean of four measurements ± standard deviation.

Figure 3 represents *L. brevis* CD2 survival in three film batches over a storage period of 48 weeks at the temperature of +2–8 °C and +23–25 °C. For the three batches, after film preparation, the mean value of *L. brevis* CD2 survival was 86%. In fact, for the batches I, II and III, viable bacterial cells declined from 9.21 ± 0.25 log CFU (*L. brevis* CD2 loaded on the film surface corresponding to 10 mg of probiotic) to 8.20 log CFU (1.01 log cell lost), 7.31 log CFU (1.90 log cell lost) and 7.61 log CFU (1.60 log cell lost), respectively. Storage conditions influenced *L. brevis* CD2 survival. Indeed, bacterial cell counts were significantly affected by both temperature and time of storage (*p* = 0.001 and *p* < 0.0001, respectively). Indeed, for all three batches, a progressive decrease of *L. brevis* CD2 survival over time was observed, but a storage temperature of +2–8 °C was preferable with respect to +23–25 °C. In batch I, bacterial viable cells declined from 8.20 log CFU to 4.92 log CFU when the buccal films were stored at +2–8 °C (3.28 log cell lost; 60% survival) and to 4.22 log CFU when the buccal films were stored at +23–25 °C (3.98 log cell lost; 51% survival) for 48 weeks. In batch II, bacterial viable cells declined from 7.31 log CFU to 3.37 log CFU (3.94 log cell lost; 46% survival) at a storage temperature of +2–8 °C and to 3.13 log CFU at a storage temperature of +24 °C (4.18 log cell lost; 43% survival). In batch III, viable bacterial cells declined from 7.61 log CFU to 6.02 log CFU (1.59 log cell lost; 79% survival) at a storage temperature of +2–8°C and to 5.35 log CFU at a storage temperature of +23–25°C (2.26 log cell lost; 70% survival). Although buccal films showed a significant decrease of *L. brevis* CD2 viability over 48 weeks of storage, at the end of the storage time, the mean values of *L. brevis* CD2 survival were still acceptable, being 62% and 55% for films stored at +2–8°C and +23–25°C, respectively. With concern for the microbial contamination, no bacteria or fungi were detected on NA and SDA plates, revealing that no microbial contamination occurred during film preparation.

### 3.5. Arginine Deiminase Activity in Buccal Films

**Figure 4:**
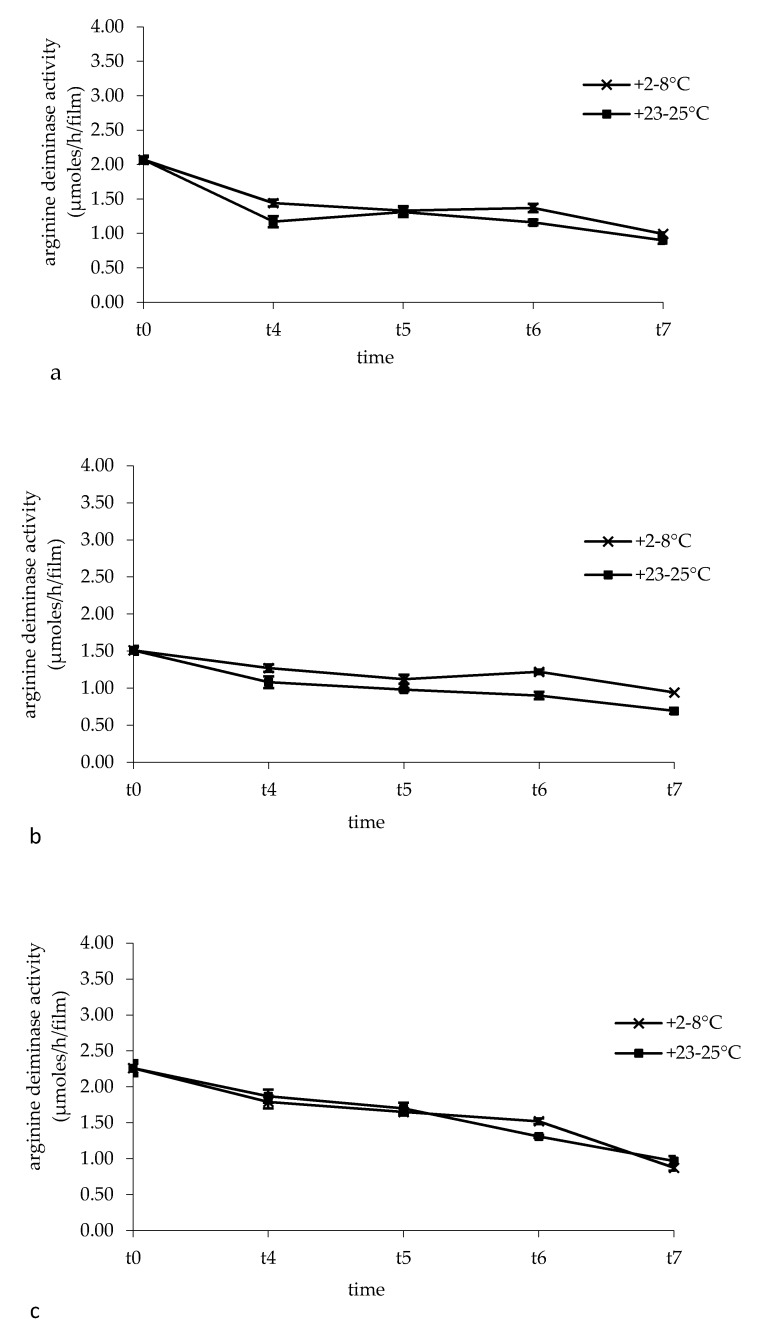
Arginine deiminase activity in the buccal films during the storage period of 48 weeks at the temperatures of +2–8 °C and +23–25 °C. t0: time zero; t4: 12 weeks; t5: 24 weeks; t6: 36 weeks; t7: 48 weeks. (**a**) Batch I; (**b**) batch II; (**c**) batch III. Data are expressed as mean of three measurements ± standard deviation.

Figure 4 shows the arginine deiminase activity of *L. brevis* CD2 monitored during 48 weeks of storage of the films at the temperature of +2–8 °C and +23–25 °C. Analyzing the influence of storage conditions, we found out that both temperature and time of storage significantly affected the enzymatic activity. In particular, storage temperature influenced the deaminase activity of films of batches I and II, but not batch III (*p* < 0.0001 for batches I and II, *p* = 0.8809 for batch III), while the time of storage correlated to a significant decrease of such activity in all three batches (*p* < 0.0001 for all batches). However, a good arginine deiminase activity was retained over a storage period of 48 weeks of buccal films, being the mean value of 1.95 µmoles/h/film at time zero and 0.93 µmoles/h/film and 0.85 µmoles/h/film when films were stored at +2–8°C and +23–25°C, respectively. 

### 3.6. In Vitro Residence Time and Mucoadhesive Ability of Buccal Films

In vitro residence time was evaluated in order to assess the ability of HPMC films containing probiotics to adhere to the buccal mucosa. Films adhered to the pig esophageal mucosa for 120 ± 7 min as indicated by the presence of a solid hydrated film; after this period a viscous gel appeared. The gel remained in situ until 20 ± 1 or 24 ± 2 h when the loaded face of the film was exposed to the medium or attached to the mucosa, respectively. These results were in agreement with our previous published data [39], where a similar residence time was observed for HPMC-based films. Moreover, the presence of sugars in *L. brevis* CD2 peptidoglycan can contribute to improving the mucoadhesive properties and consequently to increasing the residence time [37]. Mucoadhesive ability was measured with an adapted tensiometer and results showed that the detachment forces were 60 ± 1 × 10^−5^ N and 50 ± 2 × 10^−5^ N, when the loaded and unloaded face of the film were exposed to the mucosa, respectively. The highest mucoadhesive ability of the loaded face of the film favors its longer residence time with respect to the unloaded face.

### 3.7. In Vitro Release of Lactobacilli 

**Figure 5:**
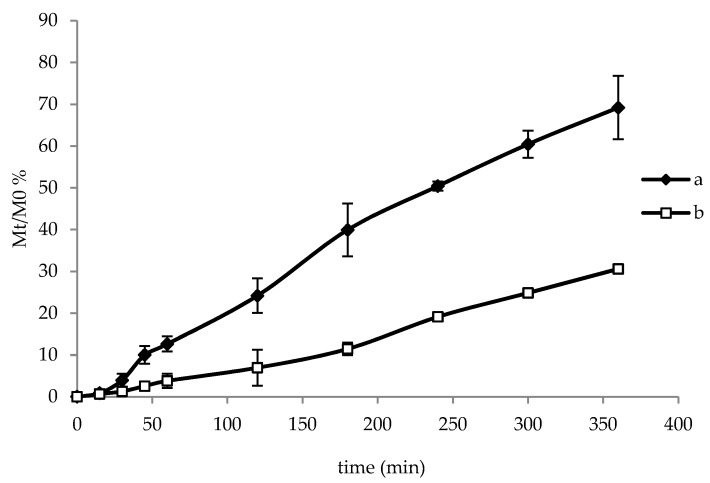
Lactobacilli release in phosphate buffer at pH 6.8 over time: (**a**) loaded face towards the medium; (**b**) loaded face towards the glass slide. Data are expressed as mean of three measurements ± standard deviation.

The release of *L. brevis* CD2 in phosphate buffer at pH 6.8 was measured by means of turbidimetric analysis using spectroscopy at 600 nm and the percentage of lactobacilli released was plotted over time (Figure 5). The test was carried out by exposing the loaded face of film towards the release medium or the glass slide. In both cases a prolonged release of lattobacilli was observed, as a consequence of film hydration that created a viscous gelled state (as also observed in Section 3.3 and Section 3.7), able to control lattobacilli release over time. When the loaded surface was exposed towards the medium (Figure 5a), 70% of lattobacilli were released after 360 min, while when the loaded face was attached towards the glass slide (Figure 5b), a lower release of lattobacilli (about 30%) was observed (*p* < 0.05). After 360 min, bacterial viable cells released from films were equal to 2.20 ± 0.10 and 5.05 ± 0.32 log CFU when the unloaded and loaded face was exposed towards the medium, respectively. These values correspond to 28% and 65% of the mean log CFU loaded on the film, thus demonstrating a good correlation with turbidimetric data. This result suggests that the film is able to deliver viable lattobacilli inside the buccal cavity or towards the mucosa by just changing the application side.

## 4. Discussion

A modified casting method was used in order to produce HPMC-based films able to deliver viable and metabolically active *L. brevis* CD2 in the oral cavity or towards the buccal mucosa, depending on the application side of the film. The modified casting method provided reproducible and uniform films in terms of thickness, weight, and water content. 

Probiotics should be handled and processed carefully in order to prevent being killed or becoming ineffective. During processing, probiotics should avoid high shear mixing, high temperatures, high pressures, high moisture and contact with solvents such as ethanol. In fact, the technological approach used in this study avoids the use of environmental and mechanical conditions, which can compromise the viability and functional activity of the probiotic strain loaded on the films. In particular, after casting the polymeric solution on the appropriate support, the drying process was performed in two steps. Firstly, drying at high temperature (50 °C) was performed in order to obtain films with minimal moisture content and able to entangle freeze-dried probiotics when sprayed on their surface. Secondly, after probiotic deposition, complete drying was achieved at room temperature until the film reached a constant weight. This procedure assures the avoidance of high temperatures and, at the same time, the loading of probiotic bacteria only on one side of the film. The compatibility of the technological process with the probiotic strain was demonstrated by the good bacterial viability after film production. A moderate loss (mean 1.50 log CFU with a mean viability of 84%) was observed probably due to both the loading process of bacteria on the film surface and to the drying step. Moreover, a good microbiological stability during the storage period, especially in refrigerated conditions, was also observed. In fact, *L. brevis* CD2 survival in buccal films stored for 48 weeks at +2–8 °C and +23–25 °C was 62% and 55%, respectively, confirming the achievement of the first objective of the study. Even more important are the results relative to the preservation of arginine deiminase activity. As reported in the introduction section, arginine deiminase plays a crucial role in reducing inflammation associated with oral disturbances and the preservation of its activity is a prerequisite for a successful probiotic treatment. Enzymatic activity was maintained until 48 weeks of storage, confirming the suitability of this technological approach.

As regards to the functional properties, buccal films showed a good ability to hydrate, in virtue of the presence of many hydrophilic groups on HPMC polymeric chains able to favour the uptake of water [40]. Moreover, HPMC is a well-known hydrophilic cellulose derivative widely used to form swellable-soluble matrices, stable over the pH range 3.0–11.0 and resistant to enzymatic degradation [41]. Water-uptake is important in order to achieve suitable adhesion of the formulation to the mucosa and to provide a three-dimensional network (hydrogel system) able to control *L. brevis* CD2 release. Great mucoadhesion and extended release represent key properties in order to achieve local persistence of probiotics and their metabolites in the buccal cavity. In this study, HPMC-loaded films showed good mucoadhesive ability as a consequence of interpenetration and entanglement of polymer chains into the mucus layer, as well as the hydrogen bonds established between the polymer hydrophilic groups and the hydrophilic groups of mucus [39,42]. The highest residence time and mucoadhesive ability were obtained when the loaded face of the film was attached to the mucosa: the presence of sugars in the *L. brevis* CD2 peptidoglycan layer may encourage the formation of other hydrogen and van der Waals bonds, thus increasing the film residence time on porcine mucosa and improving the mucoadhesive properties [37]. Finally, a prolonged release of viable lattobacilli was obtained when the film was attached by exposing the loaded face towards the release medium. This result suggests the possibility to apply the film with the loaded face towards the buccal cavity or the mucosa and consequently delivering viable lactobacilli and their metabolites onto the whole mucosa/buccal cavity or specifically onto one specific lesion. In addition, the proposed preparative method could represent a versatile approach, also considering that different amounts of lactobacilli can be easily loaded in the film by just changing the surface area (glass ring) on which probiotics are nebulized during the production process.

## 5. Conclusions

When buccal films are developed for the delivery of probiotic species, an adequate technological approach must be taken into account in order to guarantee bacterial survival. The present study demonstrates that *L. brevis* CD2 can be successfully loaded in buccal films by means of a modified casting-solvent evaporation method, in which no solvent except water was used, and room temperature was employed in order to complete the film drying, after loading of probiotics on one film face. This innovative procedure allowed good survival of *L. brevis* CD2 and the maintenance of arginine deiminase activity, especially when films were stored at the temperature of +2–8 °C. Finally, films showed good mucoadhesive properties and favored the prolonged release of viable lactobacilli when the loaded face was exposed to the release medium. In conclusion, the obtained data suggest that the film is able to deliver lattobacilli inside the buccal cavity or towards the mucosa by just changing the application side, according to the desired local treatment.

## Figures and Tables

**Table 1:** Film thickness values (µm) obtained from three different films (A, B and C) of each batch. Data are expressed as mean of five measurements performed at different random points ± standard deviation.

Film	BATCH I	BATCH II	BATCH III
A	88 ± 5	85 ± 5	80 ± 6
B	83 ± 6	82 ± 5	86 ± 4
C	83 ± 6	84 ± 6	85 ± 6
